# Antitumor activity of bacterial exopolysaccharides from the endophyte *Bacillus amyloliquefaciens sp.* isolated from *Ophiopogon japonicus*

**DOI:** 10.3892/ol.2013.1284

**Published:** 2013-04-03

**Authors:** YI-TAO CHEN, QIANG YUAN, LE-TIAN SHAN, MEI-AI LIN, DONG-QING CHENG, CHANG-YU LI

**Affiliations:** 1 Colleges of Pharmacy, Zhejiang Chinese Medical University, Hangzhou 310053, P.R. China; 2 Life Science, Zhejiang Chinese Medical University, Hangzhou 310053, P.R. China; 3 The Second Affiliated Hospital of Zhejiang Chinese Medical University, Hangzhou 310053, P.R. China; 4 Institute of Orthopaedics and Traumatology, Zhejiang Chinese Medical University, Hangzhou 310053, P.R. China

**Keywords:** *Ophiopogon japonicas*, *Bacillus amyloliquefaciens*, exopolysaccharides, endophytic bacteria, antitumor

## Abstract

The endophytic bacterium, MD-b1, was isolated from the medicinal plant *Ophiopogon japonicas* and identified as the *Bacillus amyloliquefaciens* sp. with 99% similarity based on the partial sequence analysis of 16S rDNA. Exopolysaccharides were extracted from the endophyte for the evaluation of its antitumor activity against gastric carcinoma cell lines (MC-4 and SGC-7901). 3-(4,5-dimethylthiazol-2-yl)-2,5-diphenyltetrazolium bromide (MTT) assays and microscopy were performed to estimate the cell viability and morphological changes of the MC-4 and SGC-7901 cells following treatment with the exopolysaccharides at 14, 22 and 30 *μ*g/*μ*l. The results revealed that the exopolysaccharides displayed concentration-dependent inhibitory effects against the MC-4 and SGC-7901 cells, with an IC_50_ of 19.7 and 26.8 *μ*g/*μ*l, respectively. The exopolysaccharides also induced morphological abnormalities in the cells. These effects indicated the the exopolysaccharides had an antitumoral mechanism of action associated with the mitochondrial dysfunction of the treated cells. This is the first study to investigate the endophytic microorganism isolated from *O. japonicas* and also the first discovery of such antitumoral exopolysaccharides derived from the genus *Bacillus*. This provides a promising and reproducible natural product source with high therapeutic value for anticancer treatment, thereby facilitating the development of new anticancer agents.

## Introduction

Despite recent advances in our knowledge of the molecular pathogenesis and targeted therapy of cancer, it remains one of the most malignant diseases threatening human health and quality of life. The World Health Organization has defined cancer as one of the top ten leading causes of mortality worldwide (1). Chemotherapy, combined with radiotherapy and surgery, is the main strategy for anticancer treatment. However, this strategy has limitations, including multidrug resistance and severe side effects in the clinical application, therefore impelling the search for new anticancer drugs with greater therapeutic efficiency or fewer side effects. Recently, natural products have gained increasing attention from a therapeutic point of view and have become the most consistently successful source of potential new drugs (2). Endophytic bacteria are one chief source of natural anticancer products, and are well-known for being producers of vast bioactive anti-cancer compounds, including anthracyclines, glycopeptides, aureolic acids, anthraquinones, enediynes, antimetabolites, carzinophilin and mitomycins (3–5).

Endophytic bacteria are beneficial microbes that reside in living plant tissues, mainly in the intercellular space and inside vascular tissues, without either doing harm to the host or providing any benefit to other microbial residents (6–8). The bacteria ubiquitously colonize and persist on the inner organs of plants, including the leaves, stems, seeds, tubers, fruits, ovules and, in particular, the roots, during their life-cycles (9–11). Although the interaction between these microorganisms and their respective host-plant is not, as yet, fully understood, progress has been made in the application of such bacteria as their metabolites have diverse biological functions. In total, >129 species representing >54 genera, including the *Bacillus*, *Pseudomonas* and *Agrobacterium* genera, have been isolated from agricultural plants and macrophytes (6,12,13). To date, an increasing number of bacterial endophytes have been idenitified in medicinal herbs commonly used as traditional Chinese medicines (14–16).

The plant *Ophiopogon japonicus* (Thunb) Ker-Gawl, an evergreen perennial medicinal herb, is widely distributed in South-East Asia, particularly in mainland China (Sichuan and Zhejiang provinces) (17). Its tuberous roots (known as *Mai-dong* in China) have been extensively used in traditional Chinese medicine to treat acute and chronic inflammatory diseases, as well as cardiovascular diseases, for thousands of years, as originally recorded by the ‘Shennong Materia Medica’ (Shen Nong Ben Cao Jing) (18–20), in the Eastern Han Dynasty of China (24–220 AD) and officially listed in the China Pharmacopeia (21). Phytochemical studies have revealed that *O. japonicus* is rich in the polysaccharides, homoisoflavonoids and saponins to which *Mai-dong’s* medicinal activities are largely ascribed (22–25). Recently, the majority of studies have focused on these bioactive compounds and their therapeutic functions, while little attention has been paid to the endophytes of *O. japonicas*, with only one study concerned with the isolation of actinomycetes from this herb (26). In the majority of plants, endophytes inhabit roots more easily than the aboveground tissues (27). In this regard, *Mai-dong*, the roots of *O. japonicas*, may be an overlooked promising niche for endophytic bacteria that warrants further investigation.

**Figure 1:**
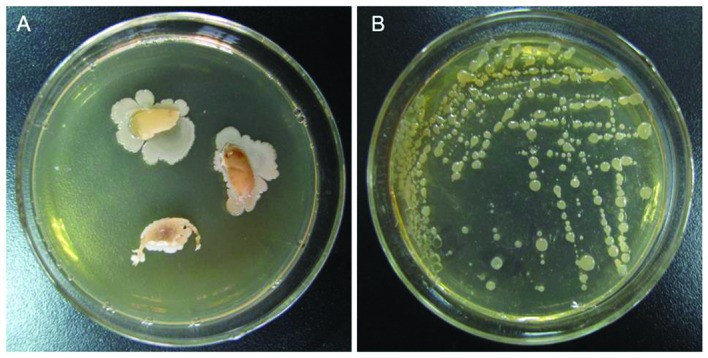
(A) Growth of endophytic bacteria from cut pieces of tuberous roots of *O. japonicas* (*Mai-dong*) on nutrient agar. (B) Sticky colonies of the endophytic bacteria growing throughout the agar medium.

In order to exploit the untapped microbial resources capable of producing useful mebabolites, we isolated a variety of endophytic fungi and bacteria in our preliminary study (28), and then discovered a Gram-positive bacteria strain with sticky colonies (Fig. 1). Further study confirmed the existence of exopolysaccharides in the colonies which might have resulted in the sticky characteristics. However, the species this strain belongs to and whether its exopolysaccharides possess useful biological activities remains unknown. Therefore, the present study was conducted to identify the endophytic bacteria strain and evaluate the bacterial polysaccharides for their antitumoral activity against gastric tumors.

## Materials and methods

### Collection and preparation of plant material

The dried tuberous roots of *O. japonicas* (*Mai-dong*) were collected from its trueborn cultivating area in Hangzhou (Zhejiang, China), and then identified and authenticated on the basis of its botanical characteristics. A voucher specimen (No. Md100912) was deposited at the College of Life Science, Zhejiang Chinese Medical University, Zhenjiang, China.

For surface sterilization, the fresh tuberous roots were thoroughly washed in running tap water to remove adhered epiphytes and soil debris, followed by washing them three times in sterile distilled water. Subsequent to being dried in sterile conditions, the root surfaces were sterilized by sequential immersion in 75% (v/v) ethanol for 5 min and 0.1% (v/v) mercury bichloride solution for 5 min. The sterilized roots were rinsed three times with sterile distilled water and excess surface sterilant was evaporated in a hot-air oven. To confirm the success of the surface sterilization, 100 *μ*l aliquots of the last washing solution were plated on Luria-Bertani (LB) media; a lack of bacterial colony growth was consequently observed on the plates.

### Isolation of endophytic bacteria

The root samples were sectioned into 4–6-mm slabs using a sterile scalpel and then transferred onto LB plates, followed by incubation at 25±2°C for 7–14 days to allow the growth of endophytic bacteria from the sections. The colonies were isolated and sequentially subcultured onto fresh LB plates for further purification. The purified colonies on the last LB plate were transferred into 5 ml liquid LB medium in conical flasks maintained at 37°C and agitated at 39 × g for 12 h. Subsequently, each suspension was centrifuged at 3,914 × g for 1 min and the deposits were dissolved in 1 ml TE buffer (1 M Tris-HCl, 0.5 M EDTA; pH 8.0). Following further centrifugation at 3,914 × g for 1 min, the deposits were collected as isolated endophytic bacteria and stored at −20°C prior to use.

### DNA extraction and identification of endophytic bacteria

The isolated endophytic bacteria were resuspended in 200 *μ*l TE buffer with 50 *μ*l lysozyme and then incubated in a water bath at 37°C for 60 min with gentle agitation. A total of 10 *μ*l proteinase K was added to the suspension, followed by incubation at 37°C for 30 min with gentle agitation. The suspension was then supplemented with 40 *μ*l 10% SDS and incubated at 37°C for 30 min with gentle agitation. Subsequent to being mixed with 100 *μ*l 5 M NaCl and 200 *μ*l 3 M sodium acetate solutions, an equal volume of chloroform:isoamylol was added and then the suspension was gently shaken. Following being left to stand at room temperature for 5 min, the mixture was centrifuged at 2,609 × g for 10 min and the supernatant was transferred into precooled isopropanol and maintained for 5 min. Following centrifugation at 3,914 × g for 10 min, the deposit was washed with 1 ml 70% ethanol and dried by evaporation at room temperature. The final deposit was dissolved with 30 *μ*l high-salt TE buffer and collected as extracted DNA for the following analysis.

The extracted DNA was electrophoresed in 1% (w/v) agarose gel, stained with ethidium bromide and UV-visualized. To determine the 16S rDNA gene sequence, PCR was conducted using the universal primers (forward, 5′-AGAGTTTGA TCCTGGCTCAG-3′; reverse, 5′-AAGGAGGTGATCCAG CCGCA-3′). PCR amplification consisted of an initial denaturation at 94°C for 5 min followed by 30 cycles (denaturation at 94°C for 1 min, annealing at 55°C for 1 min and extension at 72°C for 2 min) and a final extension at 72°C for 8 min. Amplified DNA was purified using a Takara agarose gel DNA purification kit (Takara Biotechnology Co., Ltd., Dalian, China) and sequenced by Takara Biotechnology Co., Ltd. The 16S rDNA sequence was subjected to BLAST analysis with the NCBI database and aligned by using the multiple sequence alignment program CLUSTAL W (43). A phylogenetic analysis was performed using CLUSTAL X (44) and MEGA 4.0 (45) software, based on the neighbor-joining (46), maximum-likelihood (47) and maximum-parsimony methods (48).

### Extraction and quantification of exopolysaccharides from endophytic bacteria

The isolated endophytic bacteria were incubated in liquid LB medium and agitated at 37°C for 48 h, followed by 10 min of boiling for enzyme inactivation. Following centrifugation at 1,292 × g for 30 min, the supernatant of the bacteria suspension was decolored with active carbon in a water bath at 40°C for 30 min, and then deproteinized using the Sevag method. Following subsequent centrifugation at 1,292 × g for 30 min, the supernatant was concentrated by evaporation and mixed with a 3-fold volume of 95% ethanol. The mixture was maintained overnight at 4°C, then centrifuged and its deposit dissolved with double distilled water. Following the final centrifugation, the supernatant was collected as bacteria exopolysaccharides following dialysis against double distilled water for 24 h. The content of the polysaccharides was measured using a phenol-sulfuric acid colorimetric method (49), with glucose as the reference. The concentration of polysaccharides was calculated as the polysaccharide content of extraction (mg) divided by the volume of the last liquid LB medium (3,600 ml).

### Tumor cell lines and culture condition

Human gastric carcinoma cell lines (MC-4 and SGC-7901) were provided by the Zhejiang Provincial Center for Disease Control and Prevention (Zhejiang CDC; Hangzhou, China). The MC-4 and SGC-7901 cells were cultured as described in our previous study (38). The cultured cells collected at the stage of logarithmic growth were detached using 0.25% trypsin and their viabilities were shown to be >98%, as revealed using the Trypan blue exclusion test. A suspension of each cell line containing 5×10^4^ cells was pipetted into a 96-well flat-bottomed plate and maintained in a humidified incubator at 37°C with 5% CO_2_ for 24 h.

### Antitumor evaluation of the exopolysaccharides

Samples of the extracted bacterial polysaccharides, diluted with distilled water into three concentrations (30, 22 and 14 *μ*g/*μ*l), were added to each well of each cell line respectively, followed by incubation for 18 h at 37°C with 5% CO_2_. The polysaccharide-induced cell damage was morphologically observed under a Leica DMIRE2 inverted fluorescence microscope (Leica Microsystems Corp., Bensheim, Germany). The inhibitory effects of the polysaccharides on MC-4 and SGC-7901 cell proliferation were evaluated using a 3-(4,5-dimethylthiazol-2-yl)-2,5-diphenyltetrazolium bromide (MTT) assay as described previously (38). MTT solution (10 *μ*l/well, 5 mg/ml) was added to each well and the plate was incubated for 4 h at 37°C. A total of 150 *μ*l DMSO was added to replace the supernatant in each well and the plate was gently agitated for 10 min for the dissolution of formazan crystals. The absorbance of each well at 490 nm was measured by an ELISA plate reader (Wellscan MK3; Thermo Labsystems, Helsinki, Finland). The cell viability of each treated group was calculated as the percentage of the untreated control group which was assumed to be 100%. The cytotoxicity of the polysaccharides was expressed as the IC_50_ (sample concentration causing 50% inhibition of cell proliferation) and calculated by Bliss’s method. Three replicates were conducted for the experiment.

### Statistical analysis

All measurements are expressed as the mean ± standard deviation and were subjected to one-way analysis of variance (ANOVA), followed by Fisher’s least significant difference (LSD) comparison. P<0.05 and P<0.01 were considered to indicate statistically significant differences. All analyses were performed using DPS software (Refine Information Tech. Co., Ltd., Hangzhou, China) (50).

## Results and Discussion

### Isolation and identification of endophytic bacterium from Mai-dong

**Figure 2:**
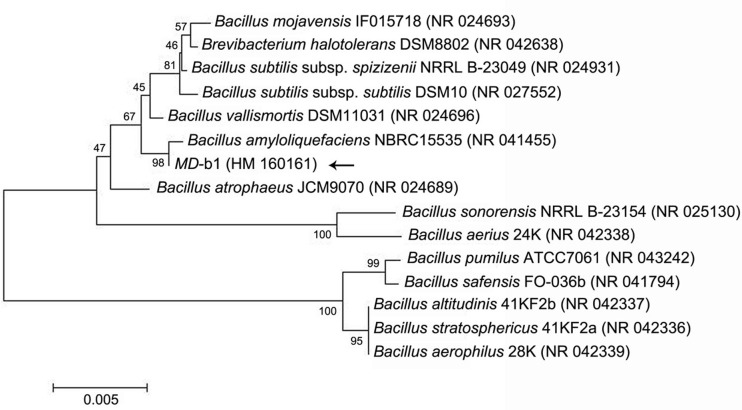
Phylogenetic neighbor-joining tree of the endophytic bacterium (MD-b1) of *Ophiopogon japonicas* tuberous root (*Mai-dong*) based on 16S rDNA gene sequences. The numbers above each branch point are confidence levels (%) generated from 1,000 bootstrap trees. Only the closest associated species with high similarities (≥97%) are indicated. Bar, 0.005 nucleotide substitutions per site.

A Gram-positive bacterium, MD-b1, with sticky colony characteristics was isolated from the inner section of *Mai-dong* and confirmed as an endophyte when all surface microbes on the plant were killed through surface sterilization. A partial sequence of the 16S rDNA gene (1,500 bp) was identified and deposited in GenBank at NCBI (Accession no. HM 160161). BLAST analysis revealed that MD-b1 belonged to the genus *Bacillus* and demonstrated the highest similarity of 99% with the *Bacillus amyloliquefaciens* strain (Accession no. NR 041455). A neighbor-joining dentrogram was constructed for the phylogenetic analysis of this endophyte bacterium, as shown in Fig. 2.

Although numerous endophytes have been isolated from traditional Chinese medicines (29), to the best of our knowledge, the present study is the first with regard to the endophytic microorganism isolated from the *O. japonicas* tuberous root (*Mai-dong*). The finding that endophytes mostly colonize the underground plant tissues, i.e., the root interior, was expected and is in agreement with previous studies (30,31). The root endophytic bacteria from the *Bacillus* genus, including the isolated MD-b1 in the present study, are capable of producing bioactive substances (e.g., mycosubtilin, iturin and surfactin) with antifungal, antibacterial and biosurfactant properties (14,32,33). Besides their application in the areas of biocontrol and agriculture, the medicinal use of metabolites from such endophytes has gained increasing attention from a pharmaceutical perspective. The only reported medicinal effect of the endophytic *Bacillus* strains was antioxidation derived from their production of extracellular enzymes, including amylase, cellulose, pectinase and xylanase (34).

### Exopolysaccharide production of MD-b1

As a major class of natural products, metabolites from microorganisms are one of the most reproducible and dependable sources for the development of ‘first-in-class’ drugs (35). The sticky characteristics of the MD-b1 colonies indicated that the MD-b1 were able to produce metabolites consisting mainly of polysaccharides. Therefore, in the present study, exopolysaccharides were extracted from this endophytic bacterium through fermentation. The content of the polysaccharides in the fermentation broth was 660 mg with a concentration of 0.22 mg/ml. No matter how many of the polysaccharides were biosynthesized by MD-b1, the large-scale production of such metabolites was always achievable using the conventional LB liquid media, indicating the potential scientific and commercial implications for its applications. Polysaccharides are a group of water-soluble bioactive compounds that have attracted considerable interest due to their wide spectrum of bioactivities and their low toxicity. The most common biological functions of polysaccharides are associated with immune system modulation, including antitumoral, antiviral and antioxidant activities. It is noteworthy that the host plants that generate the bioactive products have associated endophytes that are also able to produce the same natural products (36). Considering the antitumoral activity of the *Mai-dong* polysaccharides (37), the MD-b1-produced polysaccharides are thereby expected to have the same activity against tumors.

### Antitumoral activity of the polysaccharides derived from MD-b1

**Figure 3:**
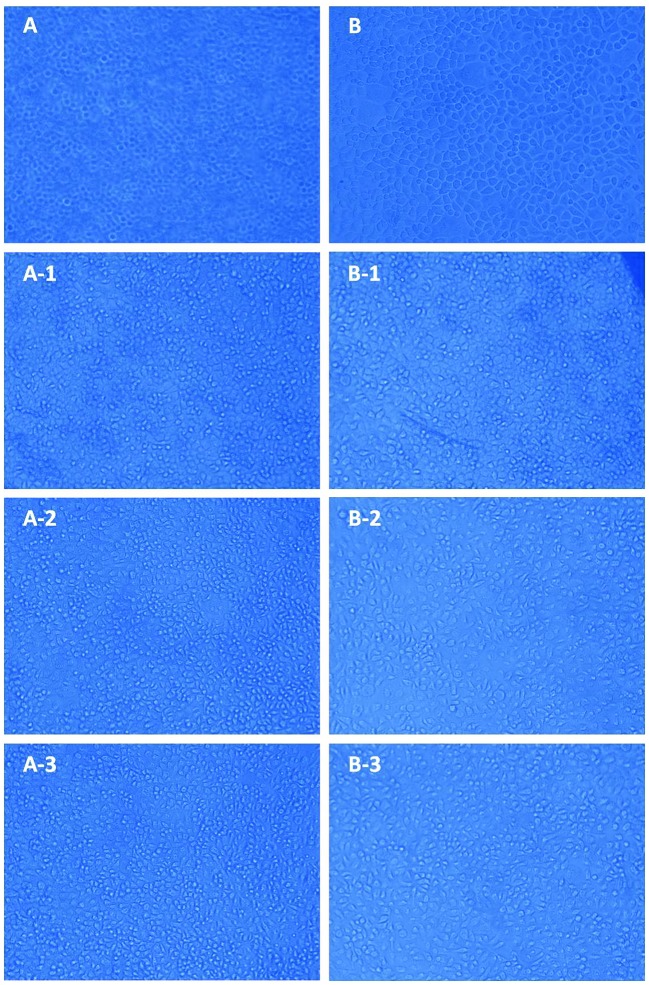
Effect of the endophytic bacterium (MD-b1)-derived exopolysaccharides on the morphology of MC-4 (A series) and SGC-7901 (B series) cells treated for 18 h. (A) Untreated MC-4 and (B) untreated SGC-7901 cells, 14 *μ*g/*μ*l polysaccharides treated MC-4 (A-1) and SGC-7901 (B-1); 22 *μ*g/*μ*l polysaccharides treated MC-4 (A-2) and SGC-7901 (B-2); 30 *μ*g/*μ*l polysaccharides treated MC-4 (A-3) and SGC-7901 (B-3).

**Figure 4:**
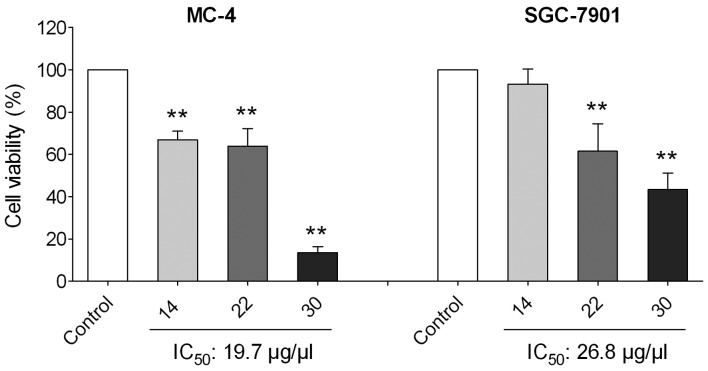
Cytotoxicity of the endophytic bacterium (MD-b1)-derived exopolysaccharides on the viability of the tumor cells (MC-4 and SGC-7901) determined by MTT assay. Results are expressed as a percentage of the untreated control group viability, and the IC_50_ for each cell line was calculated. Values are mean ± SD. ^*^P<0.05 and ^**^P<0.01 vs. untreated control group.

Light microscopy was used to observe the morphological changes to the tumor cells (MC-4 and SGC-7901). As shown in Fig. 3A and B, the untreated MC-4 and SGC-7901 cells displayed a normal shape with no apoptosis, indicating the normal condition of these cells. However, following treatment with MD-b1-derived polysaccharides at increasing concentrations (14, 22 and 30 *μ*g/*μ*l), the MC-4 and SGC-7901 cells were found to be damaged or dead with evident cell morphological abnormalities (Fig. 3A1–3 and B1–3), indicating the apoptosis-inducing effect of the polysaccharides against gastric tumor cells. Cell apoptosis may occur with cell shrinkage or collapse, membrane blebbing, boundary splitting or aggregation and nuclei condensation or nucleus fragmentation (38). Additionally, MC-4 and SGC-7901 underwent increasing morphological changes with the increasing polysaccharides concentrations, suggesting that the apoptosis-inducing effect occurred in a dose-dependent manner. The MTT assay also revealed a result consistent with this, where the significant inhibitory effect of the MD-b1-derived polysaccharides against the proliferation of the MC-4 and SGC-7901 cells was identified. As expressed by the tumor cell viability, as well as the IC_50_ estimates in Fig. 4, the polysaccharides exerted a concentration-dependent inhibitory effect against the MC-4 and SGC-7901 cells with an IC_50_ of 19.7 and 26.8 *μ*g/*μ*l, respectively. Compared with the untreated controls, significant inhibition (P<0.01) of the MC-4 and SGC-7901 cells was observed at the polysaccharide concentrations of 14–30 *μ*g/*μ*l and 22–30 *μ*g/*μ*l, respectively (P<0.01). Together, the antitumoral activity of the MD-b1-derived polysaccharides against human gastric tumor cells *in vitro* was thereby confirmed for the first time in the present study.

In recent years, gastric carcinoma has contributed to the high mortality rate of cancer worldwide, attracting increasing attention for the development of specific agents from new sources of natural products. Cytotoxicity-based screening for compounds with antitumoral activities has been previously proven to be successful in the identification of clinically applied natural anticancer products (39). In the present study, we demonstrated the prominent *in vitro* antitumoral activity of MD-b1-derived polysaccharides based on cytotoxicity assays. This demonstrated the promising prospects of such compounds in anticancer applications. The antitumor mechanism may be associated with mitochondrial dysfunction leading to mitochondrial potential loss of the tumor cells (40,41). However, the low oral bioavailability of polysaccharides, caused by their large molecular size and hydrophilic property, has limited their therapeutic applications in the clinic. However, with the co-use of absorption enhancers, such as sodium caprate, this problem may currently be overcome (23). Further chemical analysis is required to determine whether MD-b1-derived polysaccharides contain similar components to *Mai-dong* polysaccharides, since these two differently-sourced polysaccharides exhibit similar antitumoral activities. A generally accepted theory regarding this issue has suggested that the genetic recombination of the endophytes with the host may have occurred in their evolutionary period (42), resulting in the possibility that MD-b1-derived polysaccharides and *Mai-dong* polysaccharides have the same origin. If the endophytes, including MD-b1, produce the same bioactive compounds as their host plants, the fact that the rare and important natural products may be readily available and reproducible via fermentation would be noteworthy, as it may preserve the world’s ever-diminishing biodiversity by reducing the requirement for harvesting slow-growing plants. Therefore, the present study provides a promising microbial source of high-value products with significant therapeutic activities against gastric tumors, thereby facilitating the natural product identification process for new anticancer agents and benefiting anticancer therapies in practice.
